# Test-Retest Reliability of Five Times Sit to Stand Test (FTSST) in Adults: A Systematic Review and Meta-Analysis

**DOI:** 10.3390/biology10060510

**Published:** 2021-06-09

**Authors:** Laura Muñoz-Bermejo, José Carmelo Adsuar, María Mendoza-Muñoz, Sabina Barrios-Fernández, Miguel A. Garcia-Gordillo, Jorge Pérez-Gómez, Jorge Carlos-Vivas

**Affiliations:** 1 Social Impact and Innovation in Health (InHEALTH) Research Group, Faculty of Sport Sciences, University of Extremadura, 10003 Cáceres, Spain; lauramunoz@unex.es; 2 Health, Economy, Motricity and Education Research Group (HEME), Faculty of Sport Sciences, University of Extremadura, 10003 Cáceres, Spain; jadssal@unex.es (J.C.A.); mamendozam@unex.es (M.M.-M.); jorgepg100@gmail.com (J.P.-G.); jorge.carlosvivas@gmail.com (J.C.-V.); 3 Facultad de Administración y Negocios, Universidad Autónoma de Chile, Sede Talca 3467987, Chile; miguel.garcia@uautonoma.cl

**Keywords:** reliability, muscle strength, lower limbs, balance, chronic diseases

## Abstract

**Simple Summary:**

The Five Times Sit to Stand Test is a useful tool for assessing the ability to stand and sit. Its performance is dependent on lower limb muscle strength and balance control. In older adults, it is a widely used measure to assess functional mobility. However, this test is also be used in individuals with respiratory, neurological, degenerative, musculoskeletal, and other pathologies. Our study shows that the Five Times Sit to Stand Test has high reliability, defined as the overall consistency of a measure, for healthy adults or individuals with pathologies, and can be used to assess lower body strength and establish therapeutic strategies accordingly.

**Abstract:**

Functional independence in adults is conditioned by lower limb muscle strength. Thus, it seems important to assess lower limb strength using reliable and easy to reproduce measurements. The purpose of this study was to conduct a systematic review and meta-analysis to collect studies that examined the test-retest reliability of the Five Times Sit to Stand Test (FTSST) in adults. The search was conducted in PubMed, Web of Science, and Scopus databases, including all studies published up to 28 December 2020. To be included, studies had to include relative reliability scores (ICC) and maximum torque or standard error of measurements (SEM) of FTSST. A total of 693 studies were initially identified, but only 8 met the eligibility criteria and were included in the meta-analysis, covering a total of 14 groups with 400 participants. Relative inter-rater reliability results (ICC = 0.937, *p* < 0.001, *n* = 400) revealed excellent reliability of FTSST to assess sitting and standing performance, lower limbs strength and balance control. *Conclusion*: The Five Times Sit to Stand Test is a highly reliable tool for assessing lower limbs strength, balance control, and mobility in both healthy adults and those with pathologies.

## 1. Introduction

Functional mobility is necessary to perform activities of daily living (ADLs), so it is essential for the maintenance of an active lifestyle [1]. As a result of aging, decreases in muscle strength and endurance, and/or a loss in balance may occur [1,2], contributing to a decline in functional mobility and poorer health-related quality of life of individuals [3].

Several factors based on clinical assessments, such as the evaluation of physical function parameters, including the assessment of lower extremity mobility performance, balance, and loss of lower extremity strength [4], are important predictors of the development of disability in older people together with cognitive function [5] and depressive symptoms [6]. In particular, lower extremity functioning is one of the essential components for the independence of older adults in the community [7].

Muscle strength constitutes an essential quality for the mobility [8] and functional independence of people since movement is essential for the development of ADLs. Sitting and standing is considered a fundamental prerequisite for mobility [9]. The sit-to-stand movement is an activity that requires large joint torques, muscle strength of the lower extremities, sensorimotor coordination, balance, and psychological skills [10]. Thus, this action is a critical movement task performed in daily life that involves the functional ability to control the center of gravity while moving the base of support from the hips to the feet to achieve an upright posture [11].

The Five Times Sit to Stand Test (FTSST) measures the time in which an individual takes to stand up and sit down five times from a seated position [12]. To perform the test, the person must sit in a chair, with arms crossed over his chest and with his back resting on the back of the chair. Therefore, the chair should have a straight back, and it is recommended it have a height between 43–46 cm [13]. When ordered, the person should stand up completely and then return to the sitting position during 5 repetitions, performing this activity as fast as possible [14]. The FTSST is a useful, consistent, and low-cost tool for assessing the ability to stand and sit in the shortest possible time [15]. Performance on FTSST is dependent on lower limb muscle strength [16], so this test is commonly used as an indicator of lower limbs strength (particularly in adult and older populations [13,17,18]), balance control, and [16] fall risk [19]. Thus, it is a measure of physical performance, commonly used to assess functional mobility in older adults. In fact, difficulties found with FTSST (e.g., poorer performance, inability to complete the test, etc.) have been associated with abnormalities in balance and gait pattern as well as an increased risk of falls in older adults [16,20]. 

Reliability is defined as the stability or consistency of scores obtained on the same assessment test, performed on successive occasions or, in other words, the overall consistency of a measure [21]. Test reliability is essential to ensure that data provided by the measure are accurate and reflect the person’s real performance such that the changes produced in the individual are the result of interventions or other factors, and not due to measurement test errors [22]. Thus, despite its widespread use, the reliability of a test must be checked to recommend its use. Reliability can be presented as relative or absolute values [23]. On the one hand, relative reliability reports the degree to which individuals obtain similar values in a sample with repeated measurements [24]. Although different statistical parameters can be used to describe the reliability of interval or ratio measures, such as the time to complete the FTSST test, the intraclass correlation coefficient (ICC) is the most recommended and used in the literature [25]. On the other hand, absolute reliability refers to the degree to which a test measures conform from moment to moment [23]. Absolute reliability can be estimated by the standard error of measurements (SEM), the minimum detectable change (MDC), or small real difference (SRD). 

To our best knowledge, no published meta-analyses on this topic exist. Thus, this study aimed to conduct a systematic review and subsequent meta-analysis of existing test-retest reliability values of FTSST in adults with and without pathologies and to discuss potential limitations of the literature. This work may provide useful information for future protocols and optimization strategies for the development of the FTSST. 

## 2. Materials and Methods

This systematic review was performed following the Preferred Reporting Items for Systematic Reviews and Meta-analyses (PRISMA) guidelines [26] for search and study selection procedures and data collection.

### 2.1. Literature Search and Study Selection

Two investigators (L.M.-B. and J.C.-V.) independently and separately performed the search, data collection, and study analysis procedures. Both researchers independently screened and selected the titles and abstracts of the retrieved articles to check the study eligibility. Then, they carefully read and reviewed the full text of articles that met the inclusion criteria or whose eligibility was uncertain. Disagreements were resolved by consensus or consulting a third researcher.

The search was conducted in the following databases: PubMed, Web of Science (WoS), and Scopus. The following keywords linked by different Boolean operators were used: “reliability”, “ICC”, “SEM”, “Test-retest”, “SRD”, “MDC”, “chair-rise”, “sit-to-stand” and “chair-stand”. The specific search strategy was the following: ((reliability [Title/Abstract] OR test-retest [Title/Abstract] OR retest [Title/Abstract] OR ICC [Title/Abstract] OR SEM [Title/Abstract] OR SRD [Title/Abstract] OR MDC [Title/Abstract]) AND (sit-to-stand [Title/Abstract] OR chair-stand [Title/Abstract] OR chair-rise [Title/Abstract]).

After records screening, additional searches of health improvement and metasearch sources (Google/Google scholar) were conducted to identify additional potential valid publications and grey literature.

### 2.2. Eligibility Criteria

To be considered in the present systematic review and meta-analysis, studies need to meet the following eligibility criteria: (1) type of study: test-retest studies; (2) language: published in English and free full text (until 28 December 2020); (3) participants: adults aged ≥ 18 years; (4) between-session period: inter-session time should be greater than 1 day and less than 2 months difference between the first and second measurement; and (5) outcomes: studies had to report the intraclass correlation coefficient (ICC), maximum pair or SEM to describe the test-retest reliability of FTSST.

### 2.3. Statistical Analysis

Comprehensive Meta-analysis version 2 software (Englewood, NJ, USA) was used for meta-analysis, which included all eight studies included in this meta-analysis. This study focused on studies that assess the reliability of the FTSST test, both for healthy individuals and people with several pathologies such as spinal cord injury, hip osteoarthritis, chronic obstructive pulmonary disease, chronic stroke, and Parkinson’s disease. 

The reliability indicators used were ICC, SEM, and MDC. The ICC was used as the main reliability indicator. The ICCs included in our study are presented with a 95% confidence interval (95% CI) [27]. The scale designed by Landis and Koch [28] reliability strength thresholds was applied, as follows: 0, “poor reliability”; 0.01–0.20, “mild reliability”; 0.21–0.40, “fair reliability”; 0.41–0.60, “moderate reliability”, 0.61–0.80, “substantial reliability”; and 0.81–1.00, “very high or nearly perfect reliability”. The SEM was computed from the square root of the mean error term obtained from the analysis of variance (ANOVA). Then, it was used to calculate the minimum difference that is considered significant for a single individual. SEM was presented as a measure of sensitivity to change and was calculated as SEM = SD √*ICC* (1−*ICC*), where SD is the standard deviation of session 1 and session 2 [29]. In the original equation, MDC was determined as the 95% confidence limit of SEM [30] and was computed as MDC = 1.96 × √2 × *SEM* (22). SEM and MDC percentages represent the error of measurement in relative terms and therefore permit the comparisons between the different parameters. It was determined as SEM or MDC divided by the mean of all values [29].

The х-squared test in Cochran’s Q statistic (alpha set at 0.1) [31] and Higgins and Thompson’s I^2^ statistic were used to evaluate the heterogeneity among the included studies [32]. Random and fixed effects models were utilized to combine every study standardized effect size with a 95% CI. Egger’s weighted regression tests and Funnel plot were calculated to assess potential publication bias. A *p*-value lower than 0.1 was considered a statistically significant publication bias.

## 3. Results

### 3.1. Search Strategy

**Figure 1:**
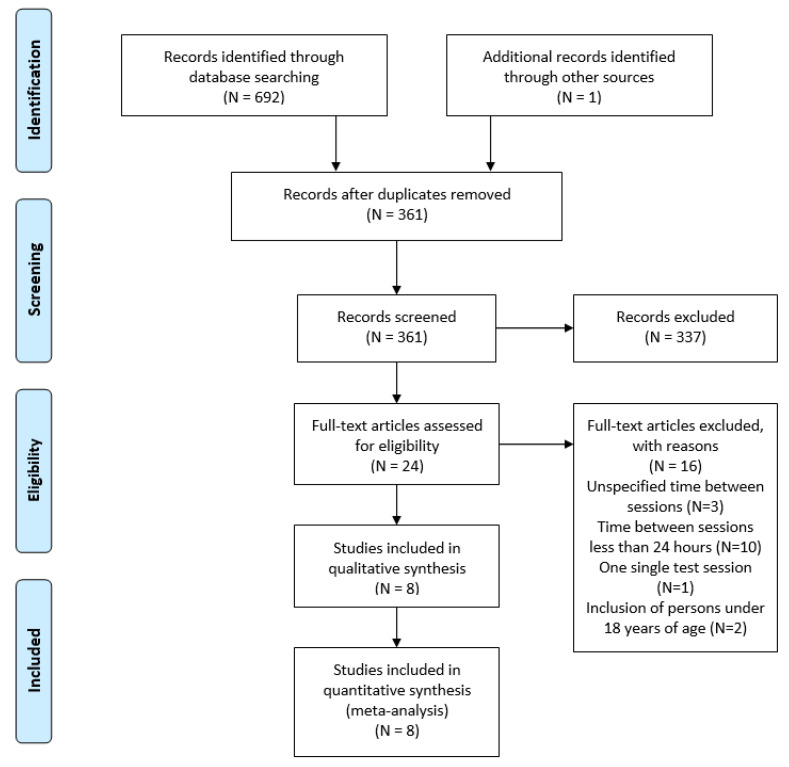
PRISMA flow diagram for study selection process.

Initially, 693 studies were identified by searching in electronic databases and additional sources. From these, 332 duplicates were identified and removed. Then, 361 studies were screened by title and abstract, excluding 337 manuscripts that were not related to the topic, were not original articles, or were not written in English. Finally, 24 articles that met the eligibility criteria were analyzed, but 16 of these studies were excluded. Three studies were removed because the time spent between sessions was not specified, ten because the between-session spent time was less than 24 h, one because it only referred to one session, and two because they included young people aged under 18 years. Therefore, eight studies were finally considered in this systematic review and meta-analysis (Figure 1).

### 3.2. Characteristics of the Included Studies

Table 1 shows the participant characteristics and reliability results of the studies included in this meta-analysis. The sample size (N) of the eight studies ranged from 15 to 80 participants, with participants aged between 21 and 94 years. All studies included participants of both sexes and the study sample consisted of participants who were disease-free [33,34] or had osteoarthritis of the hip [35], Parkinson’s disease [36,37], chronic obstructive pulmonary disease (COPD) [18], spinal cord injury [10], and chronic stroke [38]. All studies assessed the reliability of the FTSST test and reported test-retest reliability results. Some studies included reliability results for more than one group [33,35]. 

All the selected articles presented measures of force. Of these, only one [35] analyzed the effect at three different times. Only one differentiated the outcome of the ICC by sex of the participants [33]. Additionally, one study analyzed the reliability of the FTSST test with the hands placed in different positions. However, only reliability data from the test performed with the hands-on chest were selected, as indicated by the original protocol [10]. 

Substantial and very high reliability was found for all eight studies regardless of age or whether participants had any disease or pathology. The studies that assessed people with Parkinson’s disease [36,37] showed the lowest reliability.

### 3.3. Meta-Analysis

**Figure 2:**
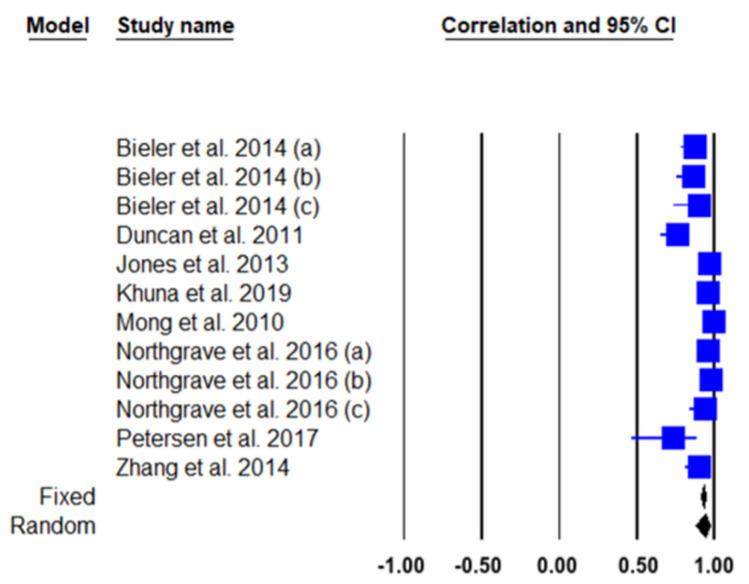
Reliability forest plot between test-retest for every study (fixed and random effects models).

We conducted a meta-analysis of the eight included studies on the test-retest reliability of FTSST. The total of the eight studies included in this meta-analysis represented the 12 analyzed groups reporting results on FTSST test-retest reliability, representing a total of 400 participants assessed. Table 2 and Figure 2 show the results of the FTSST test-retest reliability meta-analysis and the corresponding forest plot of the analyzed studies with 95% CI, respectively. The results showed significant correlation coefficients for both fixed (ICC = 0.928; *p* < 0.001) and random effects (ICC = 0.937; *p* < 0.001), with a significant heterogeneity of Q = 121.5; I^2^ = 90.94%; *p* < 0.001; and 95% CI.

Figure 2 allows a quick comparison between correlation coefficients for both fixed and random effects.

#### Publication Bias

**Figure 3:**
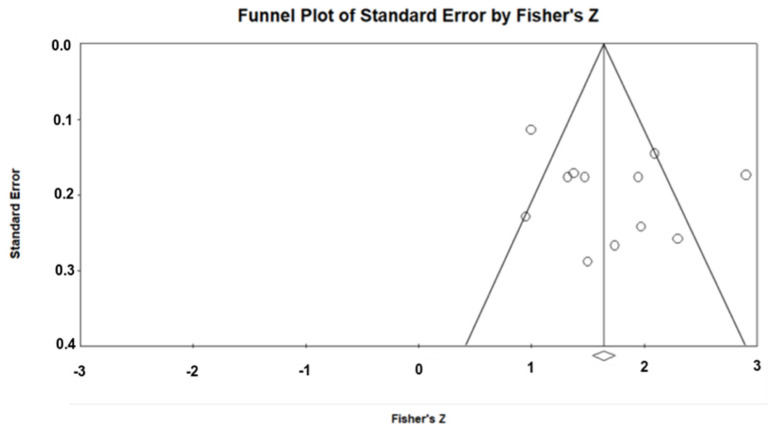
Funnel Plot of Standard Error.

Figure 3 shows the funnel plot for FTSST test-retest studies. Egger’s test was applied to assess the skewness of the funnel plot. Egger’s test outcomes for test-retest reliability of the FTSST test were not significant (*p* = 0.19 and *p* = 0.38), indicating no publication bias.

## 4. Discussion

The present review and subsequent meta-analysis explored the existing test-retest reliability of FTSST as an objective and useful assessment of lower extremities strength, balance control and fall risk.

Eight studies of healthy individuals and with different pathologies were identified for this meta-analysis: healthy individuals [33,34], individuals with hip osteoarthritis [35], Parkinson’s disease [36,37], COPD [18], spinal cord injury [10], and chronic cerebrovascular accident [38]. In this meta-analysis, a total of 12 groups were considered and a total of 400 participants were evaluated.

Studies that reported reliability of the FTSST test were analyzed. Our review suggests that studies had good to excellent reliability in the assessment of lower limbs strength (ICC = 0.74–0.99). Our results also support the assertion that FTSST results are reliable regardless of the age of the individuals and whether they have any pathology.

The groups assessing healthy people [33,34], individuals with stroke [38], COPD [18], spinal cord injury [10], and hip osteoarthritis [35] showed very high reliability. Specifically, the two studies that assessed people with Parkinson’s disease [36,37] showed substantial reliability. The sit to stand test shows its usefulness in all the studies reviewed. Although reliability is lower in people with Parkinson’s disease, it is still a test with substantial reliability. Early detection of changes in signs/symptoms through functional measures may help to identify disease progression, helping to design appropriate treatment strategies. The sit to stand test is also useful for detecting changes in functional gait and mobility in people with Parkinson’s disease [39,40].

The FTSST test is used as a measure of sit to stand performance and influences the development of a correct gait pattern and balance [20]. In all studies reporting test values, except for the one by Petersen et al. [37], the performance was faster during the second test compared to the first test, and this could be due to familiarity with the tests. The ICCs of all included studies were high for the FTSST. These results demonstrate that sit-to-stand performance, and thus, lower limbs strength can be reliably and feasibly assessed through FTSST. These data are consistent with other studies indicating that FTSST is a valid measure of dynamic balance and functional mobility in older adults [12,41]. The Mong et al. study [38] had the highest ICC, suggesting that both balance and strength after stroke influence FTSST performance. In this regard, knee extensor strength, along with measures of static balance ability (total path length and anteroposterior path velocity), report the highest magnitude of association with time to develop FTSST [42].

Deficits in sitting and standing performance in older adults are associated with fall risk [43]. FTSST requires the coordinated functioning of multiple muscle groups of lower limbs and trunk musculature. Coordination of the trunk and lower limb muscles need to avoid losses of balance during task performance. The complex nature of the FTSST test, incorporating a bidirectional center of mass control over the base of support to prevent loss of balance, makes it a useful measure for balance impairment and fall risk in older adults too.

The SEM calculated in the studies [10,33,34,35] was low in absolute terms and expressed as a percentage of the mean FTSST. The high ICC values and low SEM and SEM% values suggest excellent levels of reliability and reproducibility of FTSST, regardless of the characteristics of individuals. In addition, the MDC value reflects the smallest change in score beyond measurement errors, i.e., a true change in performance with a 95% confidence interval [44]. The MDC data provided by the studies [10,33,34,35] ranged from 1.18 to 2.93, values indicating a low change in performance. A somewhat higher value (10.3) was reported in the study by Petersen et al. [37]. However, it is still a low value and represents a low change.

Most studies had a time interval of one week between tests. One study had an interval of 24–48 h [18], while another had two weeks and two and a half weeks [35], and another four weeks [33]. The variation in time between test-retest maintained the high ICC values. The different time intervals between the first and second sessions also maintained an improvement of results in the second session compared to the first. Both studies that maintained an interval of 24–48 h and those that maintained two weeks, two and a half weeks, or four weeks had improved results in the second session except for Petersen et al. [37]. This could be due to the different abilities of the individuals with Parkinson’s disease to stand, ambulate and respond to postural perturbation without respecting the actual movement of sitting and standing. Parkinson’s disease is characterized by primarily motor degenerative disorders and has a high clinical heterogeneity in the degree of movement impairment [45]. It is possible that over time, individuals with Parkinson’s disease use compensatory strategies in sitting and standing movement, which could reduce the expected decline in FTSST performance as the disease progresses [46]. The meta-analysis performed for the reliability of FTSST (Table 2) shows that reliability is high for all groups analyzed. The results revealed excellent consistency of FTSST for both fixed (ICC = 0.928; *p* < 0.001) and random effects models (ICC = 0.937; *p* < 0.001).

In summary, the results of this meta-analysis confirm that FTSST can provide stable results in clinical practice for a wide range of populations, which confirms that FTSST is reliable for understanding sit-to-stand performance, lower limb strength, and balance control in different populations.

Nevertheless, this study has some limitations such as the heterogeneity of the selected studies (populations, ages, groups). A subgroup analysis was not performed due to the small sample size of some population groups. Within the eight studies selected, two assessed healthy individuals, two evaluated people with Parkinson’s disease, and the rest were applied in people with different pathologies. In a literature review, publication bias must be considered in the interpretation of results. Wherever possible, measures were taken to minimize bias, including a comprehensive several electronic databases search and grey literature databases. Only studies published in English and free full-text format were considered, which may have reduced the number of studies collected. Moreover, this meta-analysis was not registered in any online repository. Further studies in people with different pathologies are needed to confirm our results.

## 5. Conclusions

The FTSST is a very reliable measure of sitting and standing performance and assessment of lower limbs muscle strength and balance, regardless of whether individuals are healthy or suffering from any disease, such as hip osteoarthritis, COPD, spinal cord injury, stroke, hip dysplasia, or Parkinson’s disease. Therefore, FTSST is a useful, low cost and reliable tool for measuring dynamic balance and functional mobility. The FTSST is a potential clinical test that should be considered and included in the health care system to detect problems and to control or monitor the evolution of patients during their rehabilitation processes.

## Figures and Tables

**Table 1:** Characteristics of the included studies.

Study, Year	Sample		Age (Years)	Time of Rest	Session 1 (Seconds)	Session 2 (Seconds)	ICC (95% CI)	SEM	SEM %	MDC	MDC %
	*n*	Type									
Bieler et al. 2014	37	HOA	68 (4)	1 wk	10.14 (2.63)	9.34 (2.61)	0.880	0.91	10.7	2.11	-
	35		68 (6)	2 wk	11.37 (3.02)	10.49 (2.70)	0.867	0.99	11	2.31	-
	15		71 (5)	2.5 wk	9.99 (2.12)	9.38 (1.94)	0.905	0.63	7.7	1.47	-
Duncan et al. 2011	80	PD	67 (9.0)	7 d	20.25 (14.12)	-	0.76	-	-	-	-
Jones et al. 2013	50	COPD	69 (10)	24–48 h	14.1 (11.5)	12.4 (10.2)	0.97 (0.95–0.99)	-	-	-	-
Khuna et al. 2019	20	SCI	51.1 (13.4)	7 d	16.2 (5.97)	15.2 (4.95)	0.962 (0.905–0.985)	1.05	-	2.93	-
Mong et al. 2010	36	CS	60.0 (4.8)	7 d	17.1 (7.5)	-	0.989–0.999	-	-	-	-
Northgrave et al. 2016	35	H	54.6 (12.1)	4 wk	T (*n* = 35): 11.40 (2.89)	T: 10.96 (2.79)	T: 0.96 (0.91–0.98)	T: 0.58	T: 5.19	T: 1.60	T: 16.09
					M (*n* = 18): 10.96 (2.86)	M: 10.61 (2.94)	M: 0.98 (0.94–0.99)	M: 0.43	M: 3.94	M: 1.18	M: 10.92
					F (*n* = 17): 11.87 (2.94)	F: 11.33 (2.67)	F: 0.94 (0.82–0.98)	F: 0.71	F: 6.12	F: 1.96	F: 16.92
Petersen et al. 2017	22	PD	71.5 (8.5)	6–8 d	12.7 (7.3)	14.1 (15.2)	0.74	-	-	10.3	-
Zhang et al. 2014	35	H	81.9 (5.5)	3–8 d	17.36 (4.87)	16.29 (4.68)	0.90	-	9.0	-	-

M: males; F: females; T: full sample; wk: weeks; d: days; h: hours; HOA: hip osteoarthritis; H: healthy; PD: Parkinson’s disease; COPD: chronic obstructive pulmonary disease; SCI: spinal cord injury; CS: chronic stroke; ICC: intraclass correlation coefficient; 95% CI: 95% confidence interval; SEM: standard error of mean; MDC: minimum detectable change.

**Table 2:** Test-retest reliability meta-analysis (95% CI) for FTSST.

Study	Sample Size	Correlation Coefficient	95% IC	z	*p*	Weight (%)	
						Fixed	Random
Bieler et al. 2014 (**a**)	37	0.880	0.778 to 0.937			8.10	7.37
Bieler et al. 2014 (**b**)	35	0.867	0.751 to 0.931			7.62	7.34
Bieler et al. 2014 (**c**)	15	0.905	0.732 to 0.968			2.86	6.44
Duncan et al. 2011	80	0.760	0.649 to 0.840			18.33	7.71
Jones et al. 2013	50	0.970	0.947 to 0.983			11.19	7.54
Khuna et al. 2019	20	0.962	0.905 to 0.985			4.05	6.83
Mong et al. 2010	36	0.995	0.990 to 0.997			7.86	7.36
Northgrave et al. 2016 (**a**)	35	0.960	0.922 to 0.980			7.62	7.34
Northgrave et al. 2016 (**b**)	18	0.980	0.946 to 0.993			3.57	6.70
Northgrave et al. 2016 (**c**)	17	0.940	0.838 to 0.979			3.33	6.62
Petersen et al. 2017	22	0.740	0.463 to 0.885			4.52	6.94
Zhang et al. 2014	35	0.900	0.810 to 0.949			7.62	7.34
**Total (fixed effects)**	**400**	**0.928**	**0.912 to 0.941**	**31.325**	**<0.001**	**100.00**	**100.00**
**Total (random effects)**	**400**	**0.937**	**0.877 to 0.968**	**9.572**	**<0.001**	**100.00**	**100.00**

95% CI = 95% confidence interval; ICC = intraclass correlation coefficient; *p* = *p*-value. (**a**,**b**,**c**): Subgroups.

## Data Availability

The datasets used during the current study are available from the corresponding author on reasonable request.
